# Image Reconstruction Based on Novel Sets of Generalized Orthogonal Moments

**DOI:** 10.3390/jimaging6060054

**Published:** 2020-06-23

**Authors:** R. M. Farouk

**Affiliations:** Department of Mathematics, Faculty of Science, Zagazig University, Zagazig 44519, Egypt; rmfarouk1@yahoo.com

**Keywords:** image reconstruction, fractional order Chebyshev moments, generalized fractional order Chebyshev moments, fractional order Laguerre moments, generalized fractional order Laguerre moments

## Abstract

In this work, we have presented a general framework for reconstruction of intensity images based on new sets of Generalized Fractional order of Chebyshev orthogonal Moments (GFCMs), a novel set of Fractional order orthogonal Laguerre Moments (FLMs) and Generalized Fractional order orthogonal Laguerre Moments (GFLMs). The fractional and generalized recurrence relations of fractional order Chebyshev functions are defined. The fractional and generalized fractional order Laguerre recurrence formulas are given. The new presented generalized fractional order moments are tested with the existing orthogonal moments classical Chebyshev moments, Laguerre moments, and Fractional order Chebyshev Moments (FCMs). The numerical results show that the importance of our general framework which gives a very comprehensive study on intensity image representation based GFCMs, FLMs, and GFLMs. In addition, the fractional parameters give a flexibility of studying global features of images at different positions and scales of the given moments.

## 1. Introduction

In this paper, we focused on the problem of image reconstruction using a set of fractional order generalized orthogonal moments that allow us to use a set of parameters for each distribution separately and then study the properties of each image. The orthogonal moments of gray-scale images were firstly studied in [1] where these orthogonal moments were able to represent digital images with no redundancy or overlap of information. Moreover, orthogonal moments are robust against well-known kind of noise and have an efficient capability of features reconstruction [2]. The orthogonal moments enable researchers to reconstruct the image from a finite set of moments, using the inverse moment transform [3].

Nowadays, image representation based on a set of fractional order orthogonal moments is presented by researchers. A set of fractional order orthogonal Chebyshev moments are used to represent gray-scale image [4]. Pattern recognition based on new fractional-order Legendre-Fourier moments is introduced in [5] and also fractional order generic Jacobi–Fourier moments for image analysis presented in [6]. The fractional order polar harmonic transforms for gray-scale and color image analysis discussed in [7]. Discrete fractional order orthogonal Chebyshev moments for image encryption and watermarking based on FCMs are investigated in [8]. Shifted Chebyshev polynomials are developed to the new family of basis functions, namely generalized shifted Chebyshev polynomials [9]. The bivariate orthogonal polynomials are used to define continuous and discrete orthogonal moments and are discussed in [10]. Only a few papers have used bivariate or multivariate orthogonal polynomials for image analysis and pattern recognition [11,12,13,14,15]. In this paper, we have introduced two generalized bivariate polynomials and we have constructed a stable and orthogonal moments GFCM and GFLMs from Generalized Fractional order orthogonal Chebyshev Polynomials (GFCPs) and Generalized Fractional order orthogonal Laguerre Polynomials GFLPs respectively. The new orthogonal GFCMs and GFLMs demonstrate a very good result as shown in our numerical computation.

The paper is organized as follows. In Section 2, we mention the Chebyshev polynomials and moments and then we introduce the proposed generalized fractional Chebyshev orthogonal functions and moments FCMs and GFCMs. In Section 3, we discuss GLPs and GLMs and we present the new GFLMs. In Section 4, we demonstrate the numerical computations of the proposed FCMs, GFCMs, GLMs, and GFLMs and the effect of the polynomials parameters in reconstructed images is declared. In addition, Central Processing Unit CPU elapsed times of different proposed algorithms are demonstrated. Finally, we summarize our proposed algorithms advantages and the future work in Section 5.

## 2. Classical Chebyshev Orthogonal Polynomials

The well-known Chebyshev polynomials $C_{n}\left(x\right)$ are defined on the interval [−1,1] [16]. The $C_{n}\left(x\right)$ is defined as the solution of the Chebyshev differential equation of the first kind and can be determined from its recurrence relation in the following formula for any order n:

(1) $$C_{n+1}\left(x\right)=2\times C_{n}\left(x\right)-C_{n-1}\left(x\right),\;n=1,\;2,\;3,\;\ldots ,$$

where $C_{0}\left(x\right)=1$ and $C_{1}\left(x\right)=x$. For most application of Chebyshev polynomials, it is necessary to define the polynomials in the interval [0, 1]. As introduced in [3], the change in variable x=2y−1 and from definition of Chebyshev polynomials $C_{n}\left(2y-1\right)$, Equation (1) can be rewritten in the form:

(2) $$C_{n+1}\left(y\right)=2\left(2y-1\right)C_{n}\left(y\right)-C_{n-1}\left(y\right),\;n=1,\;2,\;3,\;\ldots ,$$

where $C_{0}\left(y\right)=1$ and $C_{0}\left(y\right)=2y-1$. In addition, Chebyshev polynomials for a given order n in the analytic form are defined in the formula:

(3) $$C_{n}\left(y\right)=n\sum_{i=0}^{n}{\left(-1\right)}^{n-i}\frac{\left(n+i-1\right)}{\left(n-i\right)!\left(2i\right)!}y^{i},\;n=1,\;2,\;3,\;\ldots ,$$

where $C_{n}\left(0\right)={\left(-1\right)}^{n}$ and $C_{n}\left(1\right)=1$. These polynomials are orthogonal and satisfy the orthogonally condition:

(4) $$\int_{0}^{1}C_{n}\left(y\right)C_{m}\left(y\right)w(y)\;dy=h_{n},$$

with respect to the weight function $w\left(y\right)=\frac{1}{\sqrt{1-y^{2}}}$ and the square norm $h_{n}=\{\begin{array}{c} \frac{\pi }{2},\;n\neq 0\\ \pi ,\;n=0 \end{array}$.

### 2.1. Chebyshev Orthogonal Moments

For any two dimensions images $f\left(x,y\right)\in L^{2}\left(\left[0,\;1\right]\times \left[0,\;1\right]\right)$, the continuously Chebyshev moment of order k+1 can be defined as in the following formula:

(5) $$CM_{kl}=\frac{1}{h_{k}h_{l}}\int_{0}^{1}\int_{0}^{1}f\left(x,y\right)C_{k}\left(x\right)C_{l}\left(y\right)w\left(x\right)w\left(y\right)dx\;dy.$$

So, the Chebyshev moment of an image of resolution N×M, Equation (5) can be approximated in the following formula:

(6) $$CM_{kl}=\frac{1}{h_{k}h_{l}}\overset{N}{\underset{i=0}{\sum }}\overset{M}{\underset{j=0}{\sum }}f\left(i,\;j\right)C_{k}\left(x\right)C_{l}\left(y\right)\;w\left(x\right)w\left(y\right)\;i=0,\;1,\;2,\;\ldots ,\;N,\;j=0,\;1,2,\;\ldots ,\;M.$$

An approximation of the original image f(x,y) is computed from the following summation:

(7) $$\hat{f}=\overset{K}{\underset{k=0}{\sum }}\overset{L}{\underset{l=0}{\sum }}CM_{kl}C_{k}\left(x\right)C_{l}\left(y\right).$$

The fractional order orthogonal Chebyshev polynomials are used for continuous functions expansion in [6] and used to represent digital images by [7]. By using the transformation $z=1-2x^{\alpha },\;\alpha >0$ in classical Chebyshev polynomials of first kind, the FC functions are defined in the interval [0,1], that we have denoted by $FC_{n}^{\alpha }\left(x\right)=C_{n}\left(1-2x^{\alpha }\right)$:

(8) $$FC_{n}^{\alpha }\left(x\right)=n\;\sum_{k=0}^{n}{\left(-1\right)}^{k}\frac{\left(n+k-1\right)}{\left(n-k\right)!\left(2k\right)!}x^{\alpha k}=\sum_{k=0}^{n}\beta_{n,k\;}x^{\alpha k},\;x\in \left[0,\;1\right],$$

where $\beta_{n,k}=n\;{\left(-1\right)}^{k}\frac{\left(n+k-1\right)!}{\left(n-k\right)!\left(2k\right)!},\;\beta_{0,k}=1,\;FC_{n}^{\alpha }\left(0\right)=1,\;\mathrm{and}\;FC_{n}^{\alpha }\left(1\right)={\left(-1\right)}^{n}$.

The $FC_{n}^{\alpha }\left(x\right)$ functions are orthogonal with weight function $w\left(x\right)=\frac{x^{\frac{\alpha }{2}-1}}{\sqrt{1-x^{\alpha }}}$ in the interval [0,1] and satisfy the orthogonal condition:

(9) $$\int_{0}^{1}FC_{n}^{\alpha }\left(x\right)FC_{m}^{\alpha }\left(x\right)w\left(x\right)dx=\frac{\pi }{2\alpha }t_{n}\delta_{nm},$$

where $\delta_{nm}$ is Kronecker delta, $t_{0}=2,$ and $t_{n}=1,\;n>1$.

The $FC_{n}^{\alpha }\left(x\right)$ can be obtained using the recursive formula as follows [4]:

(10) $$FC_{n+1}^{\alpha }\left(x\right)=\left(2-4x^{\alpha }\right)FC_{n}^{\alpha }\left(x\right)-FC_{n-1}^{\alpha }\left(x\right),\;n=1,\;2,\;\ldots ,$$

with $FC_{0}^{\alpha }\left(x\right)=1,\;\;FC_{1}^{\alpha }\left(x\right)=1-2x^{\alpha }$. The normalized fractional order Chebyshev polynomials can be defined as [4] in the following formula:

(11) $$NFC_{n}^{\alpha }\left(x\right)=\sqrt{\frac{w\left(x\right)}{t_{n}}}FC_{n}^{\alpha }\left(x\right),$$

which satisfies the orthogonally condition also:

(12) $$\int_{0}^{1}NFC_{n}^{\alpha }\left(x\right)NFC_{m}^{\alpha }\left(x\right)dx=\delta_{nm}.$$

**Figure 1:**
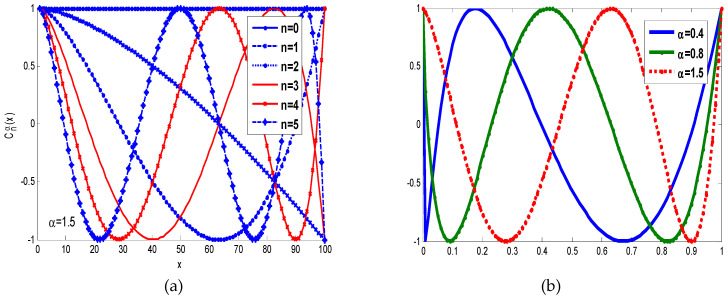

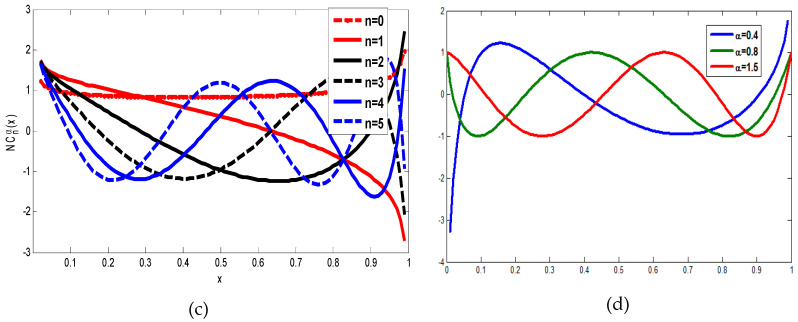
(**a**) Shows fractional order Chebyshev polynomials with different values of n, α=0.8. (**b**) Shows fractional order Chebyshev polynomials with *n* = 4 and different values of α=0.4, 0.8, 1.2, 1.6. (**c**) Normalized Chebyshev polynomials with different orders n=0, 1, 2, 3, 4, 5. (**d**) Normalized Chebyshev polynomials with n=4, α=0.4, 0.8, 1.5.

In Figure 1a, we have plotted the fractional order Chebyshev polynomials for different orders n=0, 1, 2, 3, 4, 5 and different values of α=0.4, 0.8, 1.5 in Figure 1b. In addition, we have plotted different normalized fractional order Chebyshev polynomials with different orders n=0, 1, 2, 3, 4, 5 in Figure 1c and different values of α=0.4, 0.8, 1.5 in Figure 1d.

### 2.2. Fractional Order Orthogonal Chebyshev Moments (FCMs)

For any arbitrary function f(x,y)∈[0, 1], the fractional order orthogonal Chebyshev moments of order (n+m) can be obtained from the continuous integral by the following formula:

(13) $$FC_{nm}=\int_{0}^{1}\int_{0}^{1}f\left(x,y\right)NFC_{n}^{\alpha_{x}}\left(x\right)NFC_{m}^{\alpha_{y}}\left(y\right)dxdy,$$

where $\alpha_{x},\;\alpha_{y}>0$.

For a digital image f(i, j) of resolution N×M, the fractional Chebyshev moments can be written by the formula:

(14) $$FC_{nm}=\frac{1}{NM}\overset{N-1}{\underset{i=0}{\sum }}\overset{M-1}{\underset{j=0}{\sum }}f\;\left(i,\;j\right)NFC_{n}^{\alpha_{x}}\left(x_{i}\right)NFC_{m}^{\alpha_{y}}(y_{j}),$$

where

(15) $$x_{i}=\frac{2i+1}{2N},\;y_{j}=\frac{2j+1}{2M},\;i=0,\;1,2,\;\ldots ,N,\;j=0,\;1,\;\ldots ,\;M,$$

and an approximation of the original image can obtained from the formula:

(16) $$\hat{f}=\overset{K}{\underset{k=0}{\sum }}\overset{L}{\underset{l=0}{\sum }}FC_{kl}NC_{k}^{\alpha_{x}}\left(x_{i}\right)NC_{l}^{\alpha_{y}}\left(y_{j}\right).$$

Without loss of generality, we construct a set of fractional order Chebyshev moments Equation (12), which can be used to present a digital image f(x, y). These moments for low orders can be used as low pass filter and reduce high frequencies in images. While, higher order moments store high spatial frequencies of images that correspond to the rapid changes of pixels intensities [17]. In addition, as shown in Figure 1, the fractional α moves the moment’s positions a certain position to extract local image information from a specific region of interest (ROI). In fact, the ROI can be shifted to different positions, when $\alpha_{x}<1\;,$ the ROI is shifted to the left, whereas the ROI is moved to the right when $\alpha_{x}>1$, with respect to the x-axis. The ROI is moved to the top when $\alpha_{y}<1$ and to the bottom when $\alpha_{y}>1$, along the y-axis. Eventually, Algorithm 1 describes the steps of the fractional order Chebyshev moments [18].
**Algorithm 1:** Orthogonal Fractional Chebyshev Moments [18]**Input**$f\left(x,\;y\right),\;\alpha_{x},\;\alpha_{y},\;n,\;m$**Step 1***Generate image coordinates from Equation (15)***Step 2***Generate normalized fractional order Chebyshev for*$\;n,\;\alpha_{x}$*and*$m,\;\alpha_{y}$*Equation (11)***Step 3***Calculate*$\;C_{nm}$*from Equation (14)***Output***Image moment at*n, m. 

### 2.3. Proposed Generalized Fractional Order Orthogonal Chebyshev Polynomials (GFCPs)

In this section, first, the Generalized Fractional order of the Chebyshev Polynomials (GFCPs) of the first kind has been defined and then some properties and convergence of them for our proposed algorithm have been provided. To make the generalization in Chebyshev polynomials, we have used the transformation $z=1-2{\left(\frac{x}{\eta }\right)}^{\alpha },\;\alpha ,\;\eta >0$. By substituting in Chebyshev polynomials of the first kind, we have obtained the GFCPs which are defined on the interval [0,η] and referred to it by $C_{\eta ,n}^{\alpha }\left(x\right)=C_{n}\left(1-2{\left(\frac{x}{\eta }\right)}^{\alpha }\right),$ after mathematical computation, we get GFCPs of degree nα in the formula [16,17,18,19,20]:

(17) $$C_{n,\eta }^{\alpha }\left(x\right)=\overset{n}{\underset{k=0}{\sum }}\beta_{n,k,\eta ,\alpha }x^{\alpha k},\;x\in \left[0,\;\eta \right],$$

where $\beta_{n,k,\eta ,\alpha }={\left(-1\right)}^{k}\frac{n\left(n+k-1\right)!}{\left(n-k\right)!\left(2k\right)!\;\eta^{\alpha k}},\;\beta_{0,k,\eta ,\alpha }=1,\;C_{n,\eta }^{\alpha }\left(0\right)=1,\;C_{n,\eta }^{\alpha }\left(\eta \right)={\left(-1\right)}^{n}$.

The GFCPs recurrence formula can be defined as follows:

(18) $$C_{\eta ,n+1}^{\alpha }\left(x\right)=\left(2-4{\left(\frac{x}{\eta }\right)}^{\alpha }\right)C_{\eta ,n}^{\alpha }\left(x\right)-C_{\eta ,n-1}^{\alpha }\left(x\right),\;n=1,2,\;\ldots ,$$

where $C_{\eta ,0}^{\alpha }\left(x\right)=1,\;C_{\eta ,1}^{\alpha }\left(x\right)=1-2{\left(\frac{x}{\eta }\right)}^{\alpha }$.

The GFCPs are orthogonal with respect to the weight function $w\left(x\right)=\frac{x^{\frac{\alpha }{2}-1}}{\sqrt{\eta^{\alpha }-x^{\alpha }}}$ in the interval (0, η):

(19) $$\int_{0}^{\eta }C_{\eta ,n}^{\alpha }\left(x\right)C_{\eta ,m}^{\alpha }\left(x\right)w\left(x\right)dx=\frac{\pi }{2\alpha }t_{n}\delta_{nm},$$

where $\delta_{nm}$ is Kronecker delta, $t_{0}=2,$ and $t_{n}=1$ for n≥0.

The normalized generalized fractional order Chebyshev polynomials can be defined as [18] in the following formula:

(20) $${\hat{C}}_{\eta ,n}^{\alpha }\left(x\right)=\sqrt{\frac{2\alpha w\left(x\right)}{\pi t_{n}}}C_{\eta ,n}^{\alpha }\left(x\right),$$

which satisfies the orthogonally condition also:

(21) $$\int_{0}^{\eta }{\hat{C}}_{\eta ,n}^{\alpha }\left(x\right){\hat{C}}_{\eta ,m}^{\alpha }\left(x\right)\;dx=\delta_{nm}.$$

**Figure 2:**
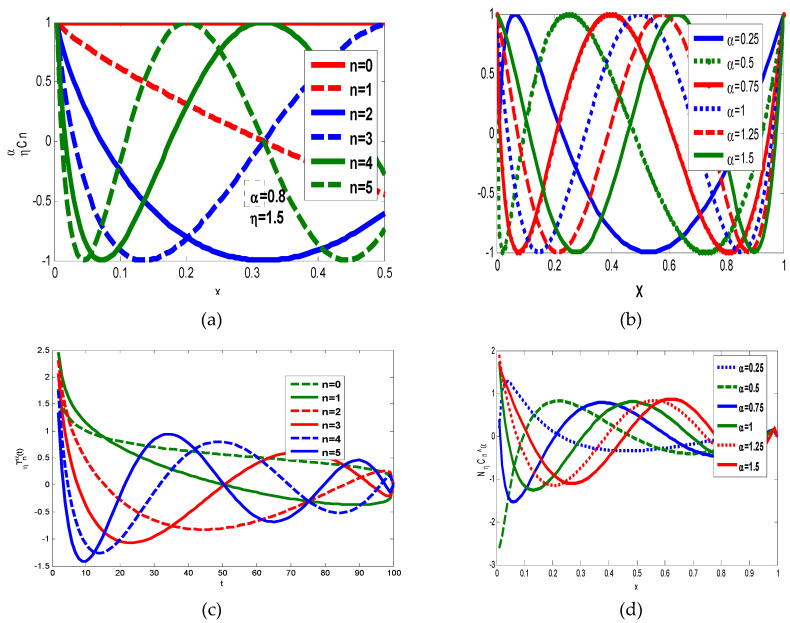
(**a**) Shows GFCPs different value of n=0, 1, 2, 3, 4, 5 and α=0.8, η=1.5. (**b**) Shows GFCPs different values of α=0.25, 0.5, 0.75, 1, 1.25, 1.5 and *n* = 5. (**c**) Normalized GFCPs different value of n=0, 1, 2,3, 4, 5 and α=1, η=1. (**d**) Shows normalized GFCPs different values of α=0.25, 0.5, 0.75, 1, 1.25, 1.5.

**Figure 3:**
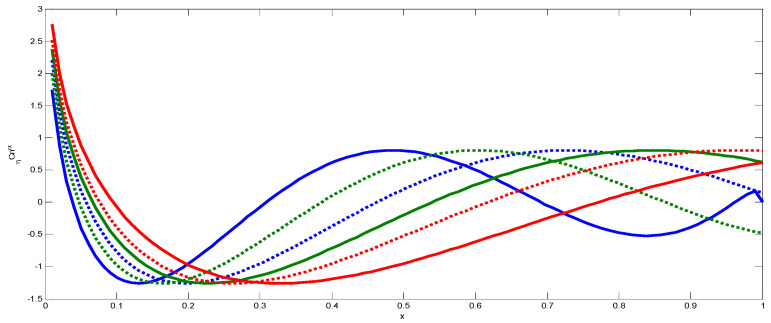
Shows different values GFCPs with different values of η=0.9, 1.5, 1.9, 2.4.

Figure 2a shows the graph of GFCPs with α=0.8 and different values of orders, and in Figure 2b, shows the graph of the GFCPs with order n=4 and various values of α. The normalized fractional order Chebyshev polynomials with different orders are displayed in Figure 2c and also the effect of the parameter η and how the moment scaled with η are displayed in Figure 2d. The generalization of polynomials is useful in moving the moments in x-direction or y-direction and scaling the moments with η values as shown in Figure 3.

### 2.4. Proposed Generalized Fractional Order Orthogonal Chebyshev Moments (GFCMs)

For any arbitrary function f(x,y)∈[0, η]×[0, η], the Generalized Fractional order orthogonal Chebyshev Moments (GFCMs) are defined by projecting the function f(x,y) onto a set of GFCPs. The GFCMs of order (*n* + *m*) for f(x,y) can be obtained from the continuous integral over all points (x,y)∈[0, η]×[0, η] by the following formula:

(22) $$G_{nm}=\int_{0}^{\eta }\int_{0}^{\eta }f\left(x,\;y\right){\hat{C}}_{\eta ,n}^{\alpha_{x}}\left(x\right){\hat{C}}_{\eta ,m}^{\alpha_{y}}\left(y\right)dxdy\;,$$

where $\alpha_{x},\;\alpha_{y}>0$. For a digital image f(i,j) of size N×M, the GFCMs can be written the formula:

(23) $$C_{\eta ,nm}^{\alpha }=\frac{1}{NM}\overset{N}{\underset{i=1}{\sum }}\overset{M}{\underset{j=1}{\sum }}f\left(i,j\right){\hat{C}}_{\eta ,\;n}^{\alpha_{x}}\left(x_{i}\right){\hat{C}}_{\eta ,\;m}^{\alpha_{y}}\left(y_{j}\right),$$

where:

(24) $$x_{i}=\frac{2i+1}{2N},\;y_{j}=\frac{2j+1}{2M},\;i=1,2,\ldots ,N;j=1,2,\ldots ,M.$$

In addition, reconstruction of the original image can be obtained from the formula:

(25) $$\hat{f}=\overset{K}{\underset{k=0}{\sum }}\overset{L}{\underset{l=0}{\sum }}C_{\eta ,kl}^{\alpha }{\hat{C}}_{\eta ,k}^{\alpha_{x}}\left(x_{i}\right){\hat{C}}_{\eta ,l}^{\alpha_{y}}\left(y_{j}\right).$$

We have constructed new shifted and scaled sets of orthogonal generalized fractional order Chebyshev moments that can represent an image at different orders and scaled to the interval [0, η]. The flexibility in choosing the parameters $\alpha_{x},\;\alpha_{y}$ and η gives the new sets of orthogonal generalized fractional moment’s prior in analysis the high and low spatial frequencies $\alpha_{x},\;\alpha_{y}$. On the one hand, as shown in Figure 2, the fractional α moves the moment’s positions to a certain position to extract local image information from a specific region of interest (ROI). In fact, the ROI can be shifted to different positions, when $\alpha_{x}<1,$ the ROI is shifted to the left, whereas the ROI is moved to the right when $\alpha_{x}>1$, with respect to the *x*-axis. The ROI is moved to the top when $\alpha_{y}<1$ and to the bottom when $\alpha_{y}>1$, along the *y*-axis. On the other hand, we can project the global information of an image to the interval [0, η]. Algorithm 2 shows the steps of GFCMs.
**Algorithm 2:** Generalized Fractional order Orthogonal Chebyshev Moments (GFCMs) [19]Input$f\left(x,y\right)\alpha_{x},\;\alpha_{y},\;\eta ,\;n,\;m$Step 1*Create image coordinates from Equation (24)*. Step 2*Compute Equation (20) for*$n,\;\alpha_{x},\eta$*and*$m,\;\alpha_{y},\;\eta$Step 3$C_{\eta ,nm}^{\alpha }$*from Equation (23)*Output *Image moment at*n, m. 

## 3. Proposed Generalized Orthogonal Laguerre Polynomials (GLPs)

Let $L_{n}^{\beta }\left(x\right),\;\beta >-1$ be the generalized Laguerre polynomials of order n. The recurrence relation of GLPs can be defined as [20,21]:

(26) $$L_{n+1}^{\beta }\left(x\right)=\frac{1}{n}\left[\left(2n+\beta -x+1\right)L_{n}^{\beta }\left(x\right)-\left(n+\beta \right)L_{n-1}^{\beta }\left(x\right)\right],\;n=1,\;2,3,\;\ldots ,$$

with $L_{0}^{\beta }\left(x\right)=1,\;L_{1}^{\beta }\left(x\right)=1+\beta -x$,

and lytic form of $L_{n}^{\beta }\left(x\right)$ is obtained as:

(27) $$L_{n}^{\beta }\left(x\right)=\overset{n}{\underset{k=0}{\sum }}{\left(-1\right)}^{k}\frac{\Gamma \left(n+\beta +1\right)}{\Gamma \left(k+\beta +1\right)\left(n-k\right)!k!}x^{k},$$

where $L_{n}^{\beta }\left(0\right)=\frac{\Gamma \left(n+\beta +1\right)}{\Gamma \left(\beta +1\right)n!}$, and Γ is Gamma function.

The generalized Laguerre polynomials are orthogonal with respect to the weight function $w_{\beta }\left(x\right)=x^{\beta }e^{-x}$ on the interval [0, ∞), and satisfies the orthogonally condition:

(28) $$\int_{0}^{\infty }w_{\beta }\left(x\right)L_{n}^{\beta }\left(x\right)L_{m}^{\beta }\left(x\right)dx=h_{n}\delta_{nm},$$

where $\delta_{nm}$ is the Kronecker delta and $h_{n}=\frac{\Gamma \left(n+\beta +1\right)}{n!}$.

**Figure 4:**
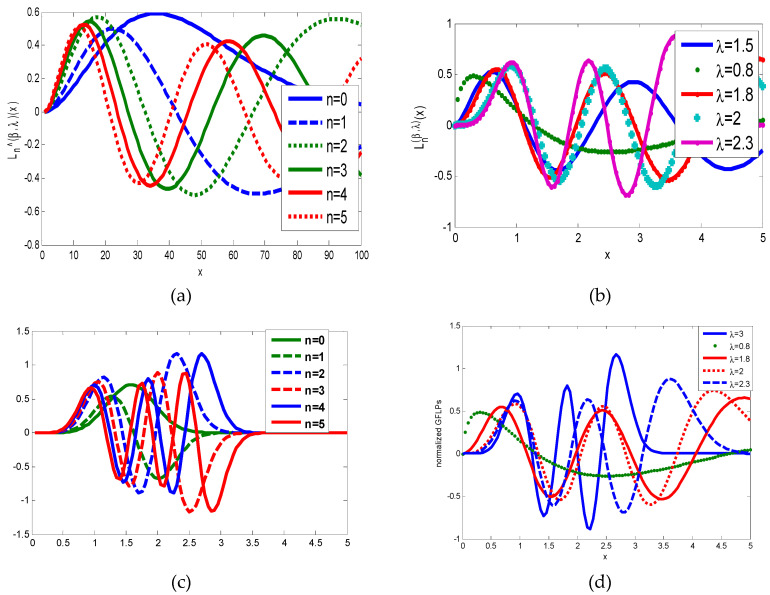
(**a**) Shows GFLPs with different orders. (**b**) Shows GFLPs with different value of λ. (**c**) Shows normalized GFLPs with different orders. (**d**) Shows normalized GFLPs with different values of λ.

Due to the generalized Laguerre polynomials, $L_{n}^{\beta }\left(x\right)$ expands rapidly with higher orders Figure 4. In numerical computation, we have used the normalized fractional order Laguerre functions ${\hat{L}}_{n}^{\beta }\left(x\right)$ defined by the formula:

(29) $${\hat{L}}_{n}^{\beta }\left(x\right)=\sqrt{\frac{w_{\beta }\left(x\right)}{h_{n}}}L_{n}^{\beta }\left(x\right).$$

### 3.1. Proposed Generalized Laguerre Orthogonal Moments (GLMs)

For any arbitrary function f(x, y)∈[0, ∞)×[0, ∞), the generalized Laguerre moments of order (n+m) can be obtained from the continuous integral by the following formula:

(30) $$L_{nm}=\int_{0}^{\infty }\int_{0}^{\infty }f\left(x,\;y\right){\hat{L}}_{n}^{\beta_{x}}\left(x\right){\hat{L}}_{m}^{\beta_{y}}\left(y\right)dxdy.$$

For a digital image f(i, j) of resolution N×M, the generalized Laguerre moments can be written by the formula:

(31) $$L_{nm}=\frac{1}{NM}\overset{N-1}{\underset{i=0}{\sum }}\overset{M-1}{\underset{j=0}{\sum }}f\left(i,j\right){\hat{L}}_{n}^{\beta_{x}}\left(x\right){\hat{L}}_{m}^{\beta_{y}}\left(y\right).$$

The reconstructed image can be obtained from the formula:

(32) $$\hat{f}=\overset{K}{\underset{k=0}{\sum }}\overset{L}{\underset{l=0}{\sum }}L_{kl}{\hat{L}}_{k}^{\beta_{x}}\left(x\right)\;{\hat{L}}_{l}^{\beta_{y}}\left(y\right),$$

where *K* and *L* are the maximum number of orders, in our computation we put *K* = *L*.

**Algorithm 3:** Generalized Laguerre Orthogonal Moments (GLMs) [21]
*Input*

$f\left(x,y\right),\;\beta_{x},\;\beta_{y},n,\;m.$

*Step 1*

*Compute*
${\hat{L}}_{n}^{\beta }\left(x\right)\;$
*for*
$n,\beta_{x}$
*and*
$m,\;\beta_{y}$
*from Equation (29)*

*Step 2*

*Calculate*
$L_{nm}$
*from Equation (31)*

*Output*
*Image moment at*n, m. 

### 3.2. Proposed Generalized Laguerre Fractional Order Orthogonal Polynomials (GLFPs)

The generalized fractional order Laguerre polynomials have been introduced in [21,22]. The (FGLPs) can be defined by substitute $t=x^{\lambda },\;\lambda >0$ on the generalized Laguerre polynomials. Let (FGLPs) $L_{n}^{\beta }\left(x^{\lambda }\right)$ be denoted by $L_{n}^{\beta ,\lambda }\left(x\right)$, by substituting in generalized Laguerre polynomials, we obtain the following recurrence relation:

(33) $$L_{n+1}^{\beta ,\lambda }\left(x\right)=\frac{1}{n}\left[\left(2n+\beta -1-x^{\lambda }\right)L_{n}^{\beta ,\lambda }\left(x\right)-\left(n+\beta \right)L_{n-1}^{\beta ,\lambda }\left(x\right)\right],\;n=1,2,\;\ldots ,$$

where $L_{0}^{\beta ,\lambda }\left(x\right)=1,\;L_{1}^{\beta ,\lambda }\left(x\right)=1+\beta -x^{\lambda }$.

The analytic form of $L_{n}^{\beta ,\lambda }\left(x\right)$ of fractional order nλ is given by:

(34) $$L_{n}^{\beta ,\lambda }\left(x\right)=\overset{n}{\underset{k=0}{\sum }}{\left(-1\right)}^{k}\frac{\Gamma \left(n+\beta +1\right)}{\Gamma \left(k+\beta +1\right)\left(n-k\right)!k!}x^{\lambda k}.$$

The new set of (FGLPs) satisfying the orthogonal condition over the interval:

(35) $$\int_{0}^{\infty }L_{n}^{\beta ,\lambda }\left(x\right)L_{m}^{\beta ,\lambda }\left(x\right)w^{\left(\beta ,\lambda \right)}\left(x\right)dx=h_{n},$$

where $w^{\left(\beta ,\lambda \right)}\left(x\right)=\lambda x^{\left(\beta +1\right)\lambda -1}e^{-x^{\lambda }}$ and $h_{n}=\{\begin{array}{c} \frac{\Gamma \left(n+\beta +1\right)}{n!}\;n=m\\ 0,\;n\neq m \end{array}$.

The normalized form of the generalized orthogonal fractional order Laguerre functions is defined by the following formula [21]:

(36) $${\hat{L}}_{n}^{\beta ,\lambda }\left(x\right)=\sqrt{\frac{\lambda x^{\left(\beta +1\right)\lambda -1}\;n!\;e^{-x^{\lambda }}}{\left(n+\beta \right)!}}L_{n}^{\beta ,\lambda }\left(x\right).$$

### 3.3. Proposed Generalized Laguerre Fractional Order Orthogonal Moments (GLFMs)

Without loss of generality of Equation (35), for a digital image f(x, y) of resolution N×M, we have constructed new sets of the generalized fractional order orthogonal Laguerre moments $L_{n}^{\beta ,\lambda }\left(x\right)$ defined by:

(37) $$L_{nm}^{\beta ,\lambda }=\frac{1}{NM}\overset{N-1}{\underset{x=0}{\sum }}\overset{M-1}{\underset{y=0}{\sum }}f\left(x,y\right){\hat{L}}_{n}^{\beta_{x},\lambda }\left(x\right){\hat{L}}_{m}^{\beta_{y}\lambda }\left(y\right).$$

It is proved that, we can reconstruct an approximation $\hat{f}$ of the original image f from the following formula:

(38) $$\hat{f}=\overset{n_{max}}{\underset{n}{\sum }}\overset{m_{max}}{\underset{m}{\sum }}{\hat{L}}_{n}^{\beta ,\lambda }\left(x\right){\hat{L}}_{m}^{\beta ,\lambda }\left(y\right),$$

where $n_{max}>n;\;m_{max}>m$.
**Algorithm 4:** Generalized Laguerre Fractional Order Orthogonal Moments (FGLMs) [21]Input$f\left(x,y\right),\;\beta_{x},\;\beta_{y},\;\lambda ,\;n,\;m.$Step 1*Compute*${\hat{L}}_{n}^{\beta ,\lambda }\left(x\right)$*for*$n,\;\beta_{x},\;\eta$*and*$m,\;\beta_{y},\;\eta$Step 2*Calculate*$L_{nm}^{\beta ,\lambda }$*from Equation (37)*Output *Image moment at*n, m. 

## 4. Discussion and Numerical Results

**Figure 5:**
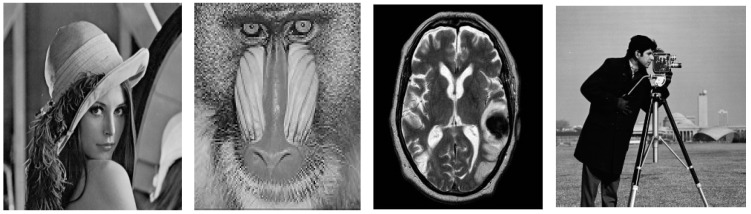
Original dataset.

To demonstrate the performance of the new introduced algorithms, FCMs, GFCMs, GLMs, and GLFMs, we completed a set of numerical experiments on dataset images, whichare displayed in Figure 5. All the algorithms and the numerical experiments are implemented and executed in MATLAB8.2 under Microsoft Windows environment using a PC with Intel Core i5 CPU 2.4 GHz and 4 GB RAM.

### 4.1. Image Representation

**Figure 6:**
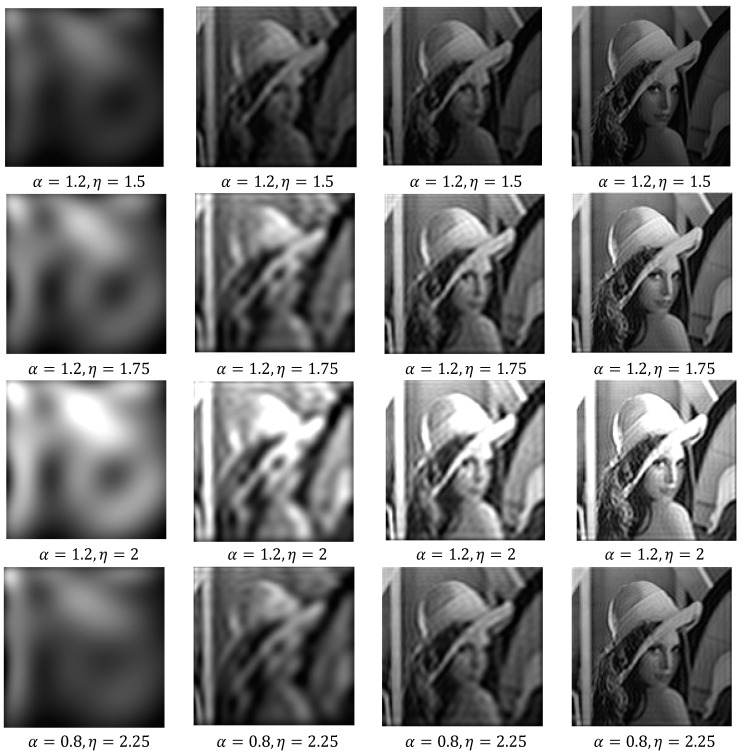
Columns 1 to 4 shows the reconstructed gray-level images with order 16, 50, 100, and 150, respectively, from GFCMs.

At first, we illustrated the influence of the parameters α and η on image reconstruction when using the generalized fractional order Chebyshev orthogonal moments. According to the constraints imposed on these parameters given by Equations (8) and (18), α,β>0. Figure 2a shows the plots of the first few orders n=0, 1, 2, 3, 4, 5 and α=0.8, η=1.5 of GFCPs, Figure 2b displays the plots of GFCPs with different values of the scale parameter α=0.25, 0.5, …,1.5, η=1.5, Figure 2c,d shows the plots of normalized GFCPs with different orders and scales. As shown in Figure 8, the fractional α moves the moment’s positions to a certain position to extract local image information from a specific region of interest (ROI). In fact, the ROI can be shifted to different positions, when $\alpha_{x}<1,$ the ROI is shifted to the left, whereas the ROI is moved to the right when $\alpha_{x}>1$, with respect to the *x*-axis. The ROI is moved to the top when $\alpha_{y}<1$ and to the bottom when $\alpha_{y}>1$, along the y-axis. On the other hand, we can project the global information of an image to the interval [0, η]. We used a gray level image of size 256 × 256 to test the effect of the parameter α and η on the reconstruction results, see Figure 6. We used mean square error (MSE) to measure the performance of the proposed GFCMs. The MSE between the original image f(x,y) and reconstructed image $\hat{f}\left(x,y\right)$ is computed from the following formula:

(39) $$MSE=\frac{1}{NM}\overset{N-1}{\underset{x=0}{\sum }}\overset{M-1}{\underset{y=0}{\sum }}{\left[f\left(x,y\right)-\hat{f}\left(x,y\right)\right]}^{2}.$$

**Figure 7:**
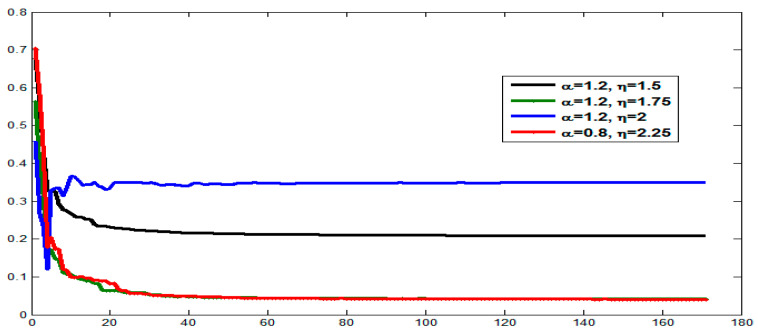
Comparison of reconstruction errors with different choices of parameters of GFCMs.

The plot of corresponding reconstruction errors is depicted in Figure 7. It can be seen that, the reconstructed image equality is increased with increasing orders and α,η>0.

**Figure 8:**
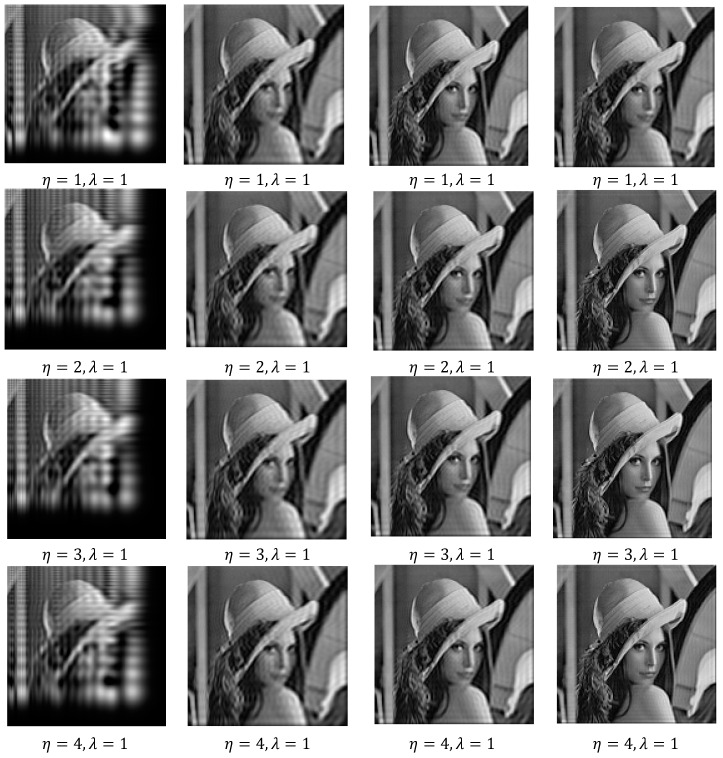
Columns 1 to 4 shows the reconstructed by using GFLMs gray-level images with order 50, 100, 150, and 300, respectively.

**Figure 9:**
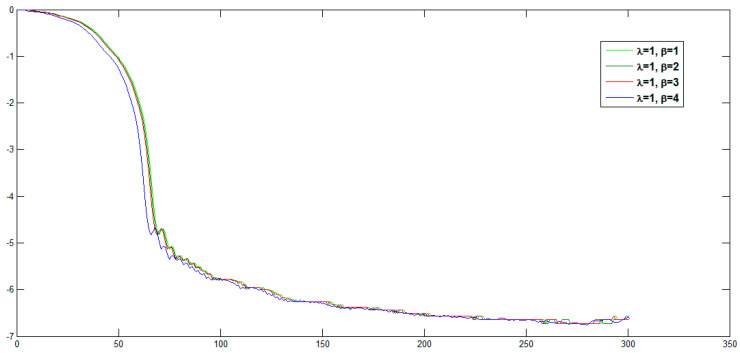
Comparison of reconstruction errors with different choices of parameters of GFLMs.

In the same manner, we have discussed the effect of the parameters η and λ on image reconstruction when using the generalized fractional order Laguerre orthogonal moments. Depending on the constraints imposed on these parameters given by Equations (26) and (33), η, λ>0. In Figure 4a, we plot GFLPs for different orders n=0, 1, 2, 3, 4, 5 and λ=1.5, in Figure 4b, we plot GFLPs with order n=5 and different values of λ=0.8, 1.5, 1.8, 2, 2.3, and in Figure 4c,d, we plot normalized GFLPs with different orders and values of λ. For image reconstruction, we used MSE Equation (36) to measure the performance of GFLMs as shown in Figure 8 columns 1 to 4. In Figure 9, we plot the MSE against number of moments. The MSE shows that with higher value of η, the error becomes higher, but it converges with higher order of moments as displayed in Figure 9.

**Figure 10:**
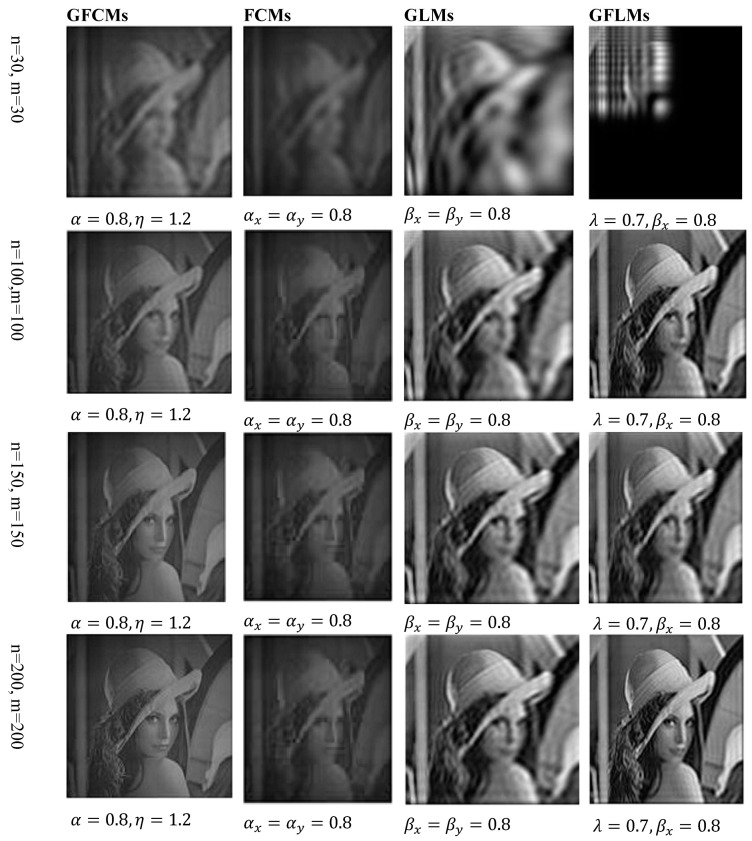
Shows the reconstructed Lena image at the same orders from the different proposed GFCMs, FCMs, Generalized Laguerre Moments (GLMs Algorithm 3 and GFLMs).

**Figure 11:**
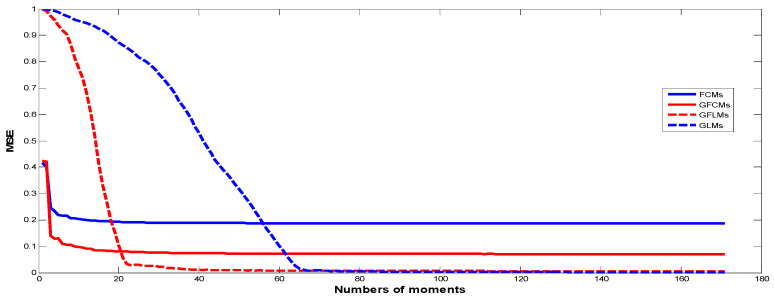
Shows the Mean Square Error MSE for the different proposed GFCMs, GLMs, and GFLMs compared with FCMs of the Lena image reconstruction error.

Finally, we compared the results that we obtained from Algorithms 2–4 with the results published recently in this field of Algorithm 1. Comparing these results, we found that the results obtained from the proposed algorithms give better results, as shown in Figure 10. We used MSE to calculate the error on image reconstruction. As is evident in Figure 10, the results obtained are better than the published results. We presented here an example for gray-level image of Lena with size 256 × 256 and we reconstructed this image from GFCMs with parameters α=0.8, η=1.2 and substituting in Equation (16) and $\alpha_{x}=\alpha_{y}=0.8$ in the case of FCMs, Equation (14). In the case of GLMs, we also used $\beta_{x}=\beta_{y}=0.8$ in Equations (29), (31), and (32). Finally, in GFLMs, we substituted by $\beta_{x}=\beta_{y}=0.8$, and λ=0.7. As shown in Figure 11, the Mean Square Error MSE of GFCMs and GFLMs give better results than FCMs and GLMs.

We reconstructed the Lena image by using the proposed algorithms for different orders n+m. In Figure 10, we displayed the reconstructed Lena image at order *n* = *m* = 30, *n* = *m* = 100, *n* = *m* = 150, *n* = *m* = 200, and *n* = *m* = 300. We observe in the numerical results that, the higher orders give better results for all algorithms except the GFLMs give reconstructed images with zeros with order higher than 200. In addition, in GFCMs, the accuracy of reconstruction is depending on α and μ, as well as the GFLMs, highly depending on β and λ.

### 4.2. Computational Time

**Figure 12:**
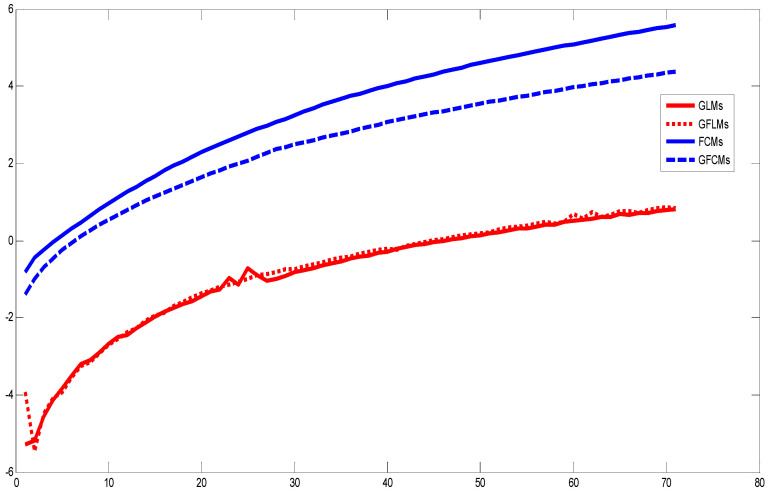
This figure displays the nature logarithm of computational time of moments obtained from different proposed GFCMs, GLMs, GFLMs, and FCM algorithms.

In order to examine the priority of the proposed novel GFCMs, GLMs, and GFLMs, we computed the computational performance of the proposed fractional order moments. Figure 12 shows the elapsed CPU times in seconds for the moment’s computation of the Lena test image, with size 256 × 256 pixels. In Figure 12, we plotted the natural logarithm of the CPU elapsed times against number of moments. According to the results presented in Figure 12, one can observe that the computation time taken by GFCMs, GFLMs is less than the CPU elapsed times taken by FCMs and GLMs. In numerical computation, we observed that the CPU elapsed times increase with increasing order of moments and also depend highly on the scale parameter in GFCMs and GFLMs η and β, respectively. The shifts parameters $\alpha_{x}=\alpha_{y}$ do not highly affect the CPU elapsed times. All curves in Figure 12 are computed with shifts parameters $\alpha_{x}=\alpha_{y}=0.8$ and η=β=1.2.

## 5. Conclusions

We introduced a new general framework for representing images based on three new sets of generalized fractional order of Chebyshev orthogonal moments (GFCMs), generalized Laguerre orthogonal moments (GLMs), and generalized fractional or orthogonal moments (GFLMs). In our numerical computations, we observed that the parameters of the generalized polynomials, and therefore, the generalized moments give a good chance to analyze the global features in the reconstructed image by moving the moments up and down in y-directions or left and right in x-direction by changing the values of $\alpha_{x},\;\alpha_{y}$, respectively. The scale and displacement parameters effect are displayed in Figure 7. The reconstructed images from the generalized moments are more accurate than normal fractional as shown in Figure 10 and also the computation time is less than the normal fractional as shown in Figure 11. As shown on Figure 11, the MES of GFCMs, GLMs, and GFLMs demonstrate the advantages of the proposed new algorithms over the FCMs introduced recently by [18]. In addition, the CPU elapsed time, as displayed in Figure 12, is less than the traditional image moments. The numerical results show that the importance of our general framework, which gives a very comprehensive study on intensity image representation based GFCMs, FLMs, and GFLMs.
